# La chirurgie du diaphragme sous aortique

**DOI:** 10.11604/pamj.2016.23.265.4212

**Published:** 2016-04-29

**Authors:** Younes Moutakiallah, Ilham Maaroufi, Mahdi Aithoussa, Mehdi Bamous, Abdessamad Abdou, Noureddine Atmani, Abdedaïm Hatim, Brahim Amahzoune, Youssef El Bekkali, Abdelatif Boulahya

**Affiliations:** 1 Service de Chirurgie Cardiovasculaire, Hôpital Militaire d'Instruction Mohammed V, Rabat, Maroc; 2 Réanimation de Chirurgie Cardiovasculaire, Hôpital Militaire d'Instruction Mohammed V, Rabat, Maroc

**Keywords:** Diaphragme sous aortique, chirurgie, récidive, Subaortic diaphragm, surgery, recurrence

## Abstract

Le diaphragme sous aortique se caractérise par une certaine latence clinique et une faible morbi-mortalité. La chirurgie reste le traitement de choix malgré un réel risque de récurrence à long terme. Nous rapportons 18 patients opérés entre Avril 1994 et Mars 2011 pour diaphragme sous aortique d’âge moyen de 18,1±9,7 ans avec 11 patients de sexe masculin. Le diaphragme était de nature fibreuse chez 13 patients et fibro-musculaire chez 5 patients. Tous les patients ont été opérés par résection de diaphragme associée à une myectomie, une plastie aortique, une fermeture de communication interventriculaire et une ligature de canal artériel perméable respectivement chez 3, 3, 2 et 2 patients. La Mortalité opératoire était nulle et sans aucun cas de trouble de conduction postopératoire. Le suivi a duré en moyenne 44,3±36,8 mois sans aucun décès tardif. Deux patients ont présenté une récidive de diaphragme qui a nécessité une réopération avec bonne évolution. La tendance actuelle dans la chirurgie du diaphragme se fait vers des interventions précoces et des résections plus extensives. Cependant, le risque de récidive impose une surveillance échographique systématique et rapprochée.

## Abstract (en)

Subaortic diaphragm is characterized by a certain clinical latency and low morbi-mortality. Surgery remains the treatment of choice despite the real risk of long-term recurrence. Our study involved 18 patients with subaortic diaphragm operated between April 1994 and March 2011. The average age was 18.1 ± 9.7 years, 11 patients were male. The diaphragm was fibrous in 13 patients and fibromuscular in 5 patients. All patients underwent diaphragm resection associated with myectomy, aortic plasty, closure of ventricular septal defect and permeable ductus arteriosus ligation in 3, 3, 2 and 2 patients respectively. Operative mortality was zero and there were no cases of postoperative conduction disorder. With a median follow-up of 44.3±36.8 months, there was no late death. Two patients had a diaphragm recurrence which required reoperation with good evolution. The current trend in diaphragm surgery is towards early interventions and more extensive resections. However, the risk of recurrence requires a systematic and close ultrasound monitoring.

## Introduction

La chirurgie du diaphragme sous aortique (DSA) est bien codifiée malgré son caractère peu commun. Il est souvent associé à d'autres malformations cardiaques comme la bicuspidie aortique (BA), la communication inter-ventriculaire (CIV), la coarctation de l'aorte (CA), le canal artériel persistant (CAP) et le canal atrio-ventriculaire (CAV). Les résultats chirurgicaux sont excellents mais au péril d'un réel risque de récurrence. Nous avons colligé entre Avril 1994 et Mars 2011, 18 patients tous porteurs d'un DSA et qui ont été opérés dans le service de chirurgie cardiovasculaire de l'hôpital militaire d'instruction Mohammed V de Rabat. A travers ce travail, nous tenons à exposer notre expérience dans cette chirurgie et ses résultats à court et à long terme pour les comparer avec la littérature.

## Méthodes

Ont été inclus tous les patients opérés pour DSA isolé ou associé à d'autres anomalies. Ont été exclu les patients porteurs de rétrécissement aortique sous valvulaire (RASV) autre que le DSA à type de tunnel sous aortique ou d'anomalies de la valve mitrale (VM) ou les RA valvaires. L'indication opératoire a été basée sur la présence de symptômes, le gradient moyen ventriculo-aortique (Gd moy) dépassant 40 mmHg, une insuffisance aortique (IA) grade 2 ou progressive sur deux examens ou la présence d'anomalies congénitales associées nécessitant une chirurgie. Les patients ont été opérés de façon élective par sternotomie médiane et sous circulation extra-corporelle (CEC) installée, après héparinisation générale (300UI/Kg), entre une canule aortique, deux canules caves et une décharge du ventricule gauche (VG) par l'apex ou la veine pulmonaire droite. La protection myocardique a été assurée par une cardioplégie cristalloïde froide antérograde (Saint Thomas) injectée directement par les ostia coronaires associée au shumway. La CEC est conduite en hypothermie modérée à 32°C avec un débit de 2 à 2,5 L/min/m^2^. L'abord cardiaque a été fait par aortotomie transversale en crosse de Hockey et réclinaison de la valve aortique (VA). La résection du DSA est faite au bistouri fin, en incisant la jonction entre le DSA et le septum interventriculaire (SIV) pour mettre en évidence un plan de clivage entre la membrane et le SIV sous la commissure et qui se poursuit par une spatule de Rob dans le sens horaire sous la sigmoïde coronaire droite (SCD), puis sous la sigmoïde coronaire gauche (SCG) jusqu'au contact de la valve mitrale antérieure (VMA), permettant ainsi une énucléation complète en une seule pièce de l'ensemble de la membrane. L'aortotomie est fermée par deux hémisurjets de fil de polypropylène 5 ou 4/0 après purge gazeuse des cavités gauches.

## Résultats

L'analyse statistique a été faite par le logiciel Software Package of Social Science (SPSS 11.5, Chicago, Illinois, USA). Les données sont exprimées en moyenne ± écart-type (extrêmes) ou en médiane si l’échantillon est hétéroclite. A L’échocardiographie, le RA était purement sous valvaire sous forme de DSA sans participation valvaire ou supravalvaire. Le siège de DSA était au niveau du SIV uniquement chez 7 patients (38,9%) et étendu à la VMA chez 6 patients (33,3%). Le DSA était de nature fibreuse chez 13 patients (72,2%) et fibro-musculaire (FM) chez 5 patients (27,8%). La valve aortique était fine chez 7 patients (38,9%) et remaniée chez 11 patients (61,1%); un patient présentait une BA (5,6%). En moyenne, l'anneau aortique mesurait 18,9±7,03 mm (17-22 mm). La moitié des patients avaient un gradient ventriculo-aortique moyen (Gd moy) qui dépassait 70 mmHg. Le VG présentait une HVG concentrique chez 15 patients (83,3%) sans retentissement sur la fonction diastolique du VG. La chambre de chasse était rétrécie chez 4 patients (22,2%) avec un cas de tunnel sous aortique et un cas de « systolic anterior motion » (SAM). Ainsi, deux patients présentaient une dilatation du VG et un seul patient avait une discrète dysfonction VG (FE = 45%). L'ETT a montré la présence respective de CIV péri-membraneuse et de CAP chez 2 patients. L'analyse de la VM a révélé un cas de remaniement valvaire et un cas d'anomalies d'insertion de cordages de la VMA. Un seul patient avait une IM grade 2 et deux patients avaient une IT grade 2. L'Euro-score moyen était de 2,1±1,7% (0,7-6,2%) (Tableau 1). Les patients ont été opérés de façon élective sauf 2 patients (11,1%) opérés en urgence différée (<48h). L'abord cardiaque a été réalisé par une aortotomie transversale en crosse de Hockey chez tous les patients et elle a été associée à une atriotomie droite parallèle au sillon atrio-ventriculaire droit chez 2 patients (11,1%) pour fermeture de CIV. L'analyse de la valve aortique a trouvé un cas de BA non sténosante et non fuyante, des sigmoïdes remaniées chez 4 patients (22,2%) et fines chez 14 patients (77,8%). L'inspection de la voie aortique a mis en évidence un DSA semi-circulaire intéressant le SIV et une partie de la VMA chez 10 patients (55,6%) et circonférentiel occupant tout le pourtour de la voie sous aortique chez 5 patients (27,8%) et sous forme de bourrelet localisé en éperon au niveau du SIV chez 3 patients (16,7%). La chambre de chasse est étroite chez 3 patients (16,7%) et normale chez le reste. Le DSA a été réséqué chez tous les patients alors que la myectomie a été faite chez 3 patients (16,7%), la VA avait nécessité un geste de désépaississement chez un patient (5,6%), une suspension commissurale chez 3 patients (16,7%). Les gestes associés ont été une fermeture de CIV chez 2 patients (11,1%) et une ligature de CAP chez 2 patients (11,1%) (Tableau 2). La Mortalité hospitalière, définie par tout décès survenu dans les 30 jours suivants la chirurgie. Elle était nulle sans décès postopératoire. La durée moyenne de la ventilation artificielle (VA) était de 7,9±5,6h (1-20h), la durée moyenne du séjour en réanimation de 38±30,7h (12-120h) et la durée moyenne du séjour postopératoire de 13,3±4,2j (8-26j). Le saignement total moyen était de 385±534,4cc (100-1375cc) avec une médiane de 375cc. Les suites étaient simples chez 15 patients (83,3%), alors que 3 patients ont présenté des complications à type de saignement (3 cas) dont un a nécessité une reprise chirurgicale, 2 cas de pneumopathie dont un a nécessité une ré-intubation pour VA transitoire (Tableau 3). Le suivi a été fait soit par consultation des dossiers de suivi postopératoire (visites médicales des contrôles systématiques) ou par contact téléphonique des patients. Le suivi a duré en moyenne 44,3±36,8mois avec une mortalité de 0% sans aucun décès tardif. Par contre, 2 patients (11,1%) ont présenté une récidive du DSA qui a nécessité une ré-opération à 38 mois et 43 mois de la 1ère intervention avec bonne évolution. Il s'agissait d'une récidive de DSA FM chez un patient et d'un bourrelet musculaire chez un autre (Tableau 3). Chez les autres patients, le contrôle échocardiographique a montré une chute du Gd moy de 68,4±13,7 mmHg à 21,6±19,6 mmHg avec une stabilité de l'IA chez les patients qui ont avait.

**Tableau 1:** Les données pré-opératoires de la population

Variable	n = 18
Age (années)	18,1±9,7 ans (5-37 ans)
Sex-ratio (male / femelle)	1,57 (11H / 7F)
Syndrome de Shone	1 (5,6%)
NYHA	1,9±0,2 (1 - 2)
Angor	1 (5,6%)
Syncope	1 (5,6%)
Poids (Kg)	47,5±19,3 Kg (15 -80 Kg)
Taille (cm)	149,7±26,7 cm (102 - 180 cm)
RCT	0,52±0,05 (0,43 - 0,65)
RRS	18 (100%)
DTDVG (mm)	44,5±4,7 mm (36 - 72 mm)
DTSVG (mm)	26,2±4,2 mm (18 - 43 mm)
FE (%)	71±4,7% (45 - 84%)
Gradient trans-aortique moyen (mmHg)	68,4±13,7 mmHg (45 - 100mmHg)
IA grade 2 - 3	4 (22,2%)
Epaisseur paroi post en diastole (mm)	11,9±3,6 mm (6 -19 mm)
Epaisseur du SIV en diastole (mm)	12,2±3,9 mm (6 - 17 mm)
Créatininémie (mg/l)	6,5±1,6 mg/l (4 - 10 mg/l)
Euro-score (%)	2,15±1,72% (0,69 - 6,18%)

**Tableau 2:** Les données opératoires de la population

Variable	n = 18
Intervention élective	16 (88,9%)
Hémodilution totale	9 (50%)
Hémodilution partielle	9 (50%)
Bicuspidie aortique	1 (5,6%)
Sigmoïdes aortiques remaniées	4 (22,2%)
Chambre de chasse étroite	3 (16,8%)
Résection du DSA	18 (100%)
Myectomie	3 (16,8%)
Plastie aortique	5 (27,8%)
Fermeture de CIV	2 (11,1%)
Ligature de CAP	2 (11,1%)
Durée de CEC (minutes)	61,1±23,9 (32 - 128)
Durée de CA (minutes)	39,5±22,3 (17 - 110)
Inotropes positives postopératoires	5 (27,8%)

**Tableau 3:** Les données postopératoires de la population

Variable	n = 18
Mortalité hospitalière	0 (0%)
Saignement total (ml)	385±534,4 (100 - 1375)
Durée de VA (heure)	7,9±5,6 (1 - 20)
Durée du séjour en réanimation (heure)	38±30,7 (12 - 120)
Durée du séjour hospitalier postopératoire (jour)	13,3±4,2 (8 - 26)
Suites simples	15 (83,3%)
Durée de suivi (mois)	44,3±36,8 (12 - 85)
Récidive	2 (11,1%)
Ré-opération	2 (11,1%)
Mortalité tardive	0 (0%)

## Discussion

L'histoire naturelle du DSA est souvent dominée par une certaine latence aussi bien sur le plan de la symptomatologie, la découverte et finalement le recours aux thérapeutiques. Ainsi la découverte du DSA est le plus souvent fortuite, faite dans 33,3% des cas durant la 1^er^ décade de vie avec un âge moyen de découverte de 12 ans dans notre série, chez Abid [1] il est découvert avant la 1er décade chez 82% des patients et avant l’âge de 4ans chez 34%. Selon Bernard [2], seulement 18% des patients étaient symptomatiques au moment du diagnostic du DSA contre 94% de nos patients. Ceci peut être expliqué par l’âge assez avancé de nos patients par rapport aux autres séries qui était de 18 ans versus 10ans pour Serraf [3], <12 ans pour Abid [1], 4 ans pour Ali Dodge-Khatami [4] et 6ans pour Marasini [5]. La dyspnée d'effort est le symptôme le plus fréquent, elle est retrouvée dans 55% des cas (classe II) dans la série d'Abid [1] et chez 34 malades pour Serraf [3] (classe 3-4) versus 94% de nos patients (classe 2), 39% des palpitations et 6% d'angor d'effort. Dans notre série, la répartition selon le sexe a montré une prédominance masculine (11H et 7F) avec un sex-ratio de 1,57. Cette prédominance masculine qui reste sans explication est notée chez Serraf [3] (107H et 53F), chez Abid [1] (35H et 21F) et chez Marasini [5] (26H et 19F). La présence de l'IA au moment du diagnostic, ainsi que son apparition ultérieure, a longtemps été reconnue comme commune chez les patients atteints de DSA; sa fréquence est variable allant de 29,2% [6], 36% [3] à 79,5% [1]. Dans notre série, nous avons trouvé 4 cas (22,2%) d'IA grade 2-3 et 10 cas (55,6%) d'IA grade 1. Selon la majorité des auteurs, l'indication chirurgicale est formelle pour le DSA symptomatique alors qu'en absence de symptômes, les tendances sont variables; ainsi si un gradient significatif est toujours nécessaire chez la majorité des auteurs, chez les nourrissons et les enfants une chirurgie plus rapide est prônée même pour un gradient plus bas. Comme la plupart des auteurs, la stratégie s'est appuyée chez Abid [1] sur 3 éléments essentiels: le gradient maximal (Gd max) dépassant 50mmHg, l′IA progressive et une lésion associée nécessitant une cure sous CEC. En fait, en plus de ces critères, plusieurs autres éléments sont à considérés avant de poser l'indication opératoire comme l’âge lors du diagnostic, le retentissement fonctionnel, électrique et échographique et surtout le type de RASV. Pour certains auteurs, un Gd max de 40mmHg ou même 30mmHg pour Coleman [7] est suffisant pour l'indication opératoire car le risque d'IA était plus élevé dans les formes les plus serrées. Brauner [8], en colligeant 75 patients opérés pour RASV (68 DSA et 7 tunnels), a trouvé que le groupe de DSA avec Gd moy inférieur à 40mmHg avait un taux de récidive et de progression de l'IA significativement plus bas que le groupe avec gradient plus élevé. Pour Serraf [3], les critères de l'indication chirurgicale étaient le Gd max dépassant 50 mmHg, l'IA ou la présence de symptômes. Pour Marasini [5] dont les patients étaient essentiellement des enfants, les critères étaient le Gd moy de 25mmHg ou l'IA. Dans notre série, le Gd moy était élevé avec une moyenne de 67,5mmHg (Tableau 4 et Tableau 5).

**Tableau 4:** Anomalies cardiaques associées selon les séries

Anomalies associées	Abid [30]	Serraf [29]	Marasini [30]	Notre Série
C.I.V	2	Exclue	1	2
C.I.A	0	7		0
C.A.V			4	0
Coarctation isthmique	1	26	7	0
RA valvulaire	9	24		0
RA supra-valvulaire	2	2		0
Insuffisance mitrale	5	9	12	0
Valve mitrale en parachute	2			0
RM par fusion commissurale	1	13		0
Sténose mitrale		1		0
VDDI				0
Bicuspidie aortique			9	1
Anévrysme de l'aorte ascendante				0
CAP				2

**Tableau 5:** Résultats d’échographie doppler des différentes publications

		IA			
Auteur	Gradient (mmHg)	Total	Grade I	Grade II	Grade III
Serraf [29]	80 ± 34	57	41	24	1
Abid [30]	52 (moy) 85 (max)	35	23	11	1
Hirata [11]	67,3 ± 29 (max)	31	24	7	0
Marasini [32]	39,7 (moy)	45	18	7	1
Ali dodge-Khatami [31]	52	34	8	23	3
Notre série	68,4 ± 13,7 (moy) 113,2 ± 27,8 (max)	8	4	2	2

Sur le plan du traitement chirurgical, si la résection du DSA fait l'unanimité, le débat persiste quant à l'intérêt de la myotomie ou de la myectomie. Abid [1] a réalisé 5 cas de simple résection du DSA, 27 cas de résection-myectomie et 12 cas de résection-myotomie. Le choix entre la myotomie et la myectomie s'est fait en fonction de l'importance de l'hypertrophie musculaire septale. Les résultats ont montré que le Gd max dans le groupe myectomie est moins élevé durant le suivi. En effet l'association d'une myectomie a permis selon plusieurs auteurs de réduire considérablement aussi bien le Gd moy postopératoire immédiat que le taux de récidive. Lavee [9], dans une série de 42 patients a constaté que les résultats immédiats et à long terme de l'association résection-myectomie sont meilleurs. De même, Ritter [10] selon une étude de 23 patients, a constaté au terme d'un suivi de 14 ans que le groupe résection-myectomie avait un taux significativement moindre de récidive et d'IA comparé au groupe résection simple du DSA. Marasini [5] a réalisé la myectomie pour tous ses malades. Par contre, d'autres auteurs ont remis en cause cette conclusion; comme Serraf [3] et Ali Dodge-Khatami [4] qui n'ont constaté aucune différence de Gd moy postopératoire par l'association ou non d'une myectomie. Dans la série de Hirata [6], le groupe de patients porteur d'un DSA isolé, n'avait pas de différences significatives dans les taux de récidive (23% vs 30%) ou de ré-interventions (4,7% vs 4,4%) en cas d'association ou non d'une myectomie. Cependant, chez les patients présentant des anomalies cardiaques associées, la myectomie était plus efficace. Or, plusieurs auteurs attribuent à ces gestes de résection assez étendue des troubles de la conduction postopératoire; ainsi Stellin [11] n'est pas favorable à la réalisation systématique de ces gestes. Parry [12] a constaté qu'une résection agressive avec myectomie vaste s'accompagne d'un taux de BAV complet supérieur de 14% au taux rapporté par Ali Dodge-Khatami [4], Serraf [3] et Abid [1] qui était respectivement de 1.7, 2.5 et 8.9%; il souligne ainsi le compromis entre le risque de BAV et un taux de récidive potentiellement faible (aucune récidive) associée à une myectomie étendue. Le bloc de branche gauche n'est pas exceptionnel; sa fréquence était chez Jones [13], Ashraf [14] et Moses [15] respectivement de 2/20 (10%), 7/46 (15%) et 3/31 (9,7%). Le bloc de branche droit est dû surtout au patch de fermeture de CIV. Dans notre série, nous ne rapportons aucun cas de trouble de conduction postopératoire. La mortalité hospitalière est généralement faible surtout pour les formes localisées. Dans notre série, elle était nulle comme chez Ali-Khatami [4] et Marasini [5] alors que Hirata [6] a rapporté un décès. Pour Jones [13], elle est estimée à 0-4% dont la moitié survenant dans les 6 premiers mois. Pour Abid [1], le décès est survenu en cas de tunnel (2 décès soit 3,5%). Serraf [3] a rapporté 2 cas de décès avec à l'analyse statistique univariée une augmentation significative du risque de décès précoce pour la classe 3-4 de la NYHA préopératoire et l’âge avancé à l'opération alors que seule la classe NYHA préopératoire ressort comme facteur de risque indépendant à l'analyse multivariée. La mortalité à long terme est également faible; Abid [1] et Marasini [5] n'ont rapporté aucun décès tardif contre un décès chez Hirata [6] et Ali-Dodge-Khatami [4] et 4 chez Serraf [3]; nous ne rapportons aucun décès tardif après une période de suivi d'environ 4 ans en moyenne.

La récidive du RASV durant le suivi est définie par l'apparition à l’échographie de lésions obstructives ou l'apparition d'un Gd max supérieur à 30mmHg et qui étaient absents lors du contrôle échographique postopératoire immédiat. Le délai de récidive est défini par le temps séparant la date de l'intervention du ^er^contrôle échographique qui a détecté la récidive. Abid [1] a rapporté 3 cas de récidive dont deux en diaphragme et un en bourrelet FM; les deux patients avec DSA avaient été traités par résection-myotomie et le patient avec bourrelet par d'une myectomie large; l'exploration échographique a révélé chez les patients avec DSA une récidive d'un néo-diaphragme circonférentiel qui était absent lors du contrôle échographique précoce. Marasini [5] a décrit 6 cas de récidive dont 1 cas avec RA associé, 3 cas associés à d'autres anomalies de la voie de chasse soit non détectées ou non corrigées lors de la 1^ère^ intervention et 2 cas de récidive isolée. Hirata [6] a rapporté durant un suivi moyen de 6,9 ans, 26 cas (27%) de Gd max dépassant 30mmHg. Même fréquence a été décrite par Serraf [3] pour le même seuil de Gd max avec un délai moyen de réapparition du Gd max était de 3,7±3, 4 ans. L’étude statistique a mis l'accent sur un certain nombre de facteurs prédictifs de récidive du RASV et qui étaient en analyse uni-variée l'hypoplasie de l'anneau aortique, le tunnel, la CA, la myectomie et la membranectomie isolées, le gradient préopératoire élevé et le gradient postopératoire immédiat; alors qu'en analyse multi-variée les facteurs de risque indépendants de récidive étaient l'existence d'une CA et le gradient postopératoire immédiat. La résection précoce a été préconisée par plusieurs auteurs qui ont affirmé que cela réduit le taux de récidive. Toutefois, pour Serraf [3] un âge inférieur à 5ans était un facteur de risque important de récidive dans l'analyse uni-variée mais pas dans l'analyse multi-variée. Ce résultat est similaire aux conclusions de Brauner [8] et d'Ali Dodge-Khatami [4]. Selon Serraf [3], les patients avec un gradient résiduel supérieur à 30mmHg à la fin du geste devraient subir une ré-opération avec une résection sous-aortique plus agressive. Ashraf [14] a constaté que la persistance d'un gradient postopératoire supérieur ou égale à 30mmHg favorise la récidive. Les principales indications sont la persistance d'un gradient sévère, la récurrence, l'aggravation de l'IA et l'IM iatrogène. Un seul patient a été réopéré dans la série de Marasini [5]. Pour Hirata [6] les indications de la ré-opération ont été un Gd max de 50mmHg ou plus et l'HVG; 8 patients (7,5%) ont été réopérés pour RASV récurrent. Vingt patients (12,9%) chez Serraf [3] ont été réopérés ans un délai moyen de 5,3± 4,6 ans, la récidive du RASV a été la cause de ré-intervention dans 17cas, avec un Gd moy de 95±29 mmHg dont 4 cas de 2e ré-intervention. L'analyse statistique a révélé que le taux de ré-intervention a été influencé en analyse univariée par la présence d'une hypoplasie de l'anneau aortique, la présence d'une CA, un plus jeune âge à la 1 ^ère^ opération et le gradient postopératoire immédiat. En analyse multi-variée, l'existence d'une CA et le gradient postopératoire immédiat ont de nouveau été des facteurs prédictifs indépendants de ré-opération. Pour Ali Dodge-Khatami [4], une ré-opération a été nécessaire chez 11 patients (19%) à 2,6 ans (0,3-7,5 ans) après la chirurgie initiale. Dans notre série, nous avons rapporté 2 cas (11,1%) de récidive du DSA qui a nécessité une ré-opération à 38 mois et 43 mois de la 1 ^ère^ intervention avec bonne évolution. Il s'agissait d'une récidive de DSA FM chez un patient et d'un bourrelet musculaire chez un autre.

Concernant l'amélioration du statut fonctionnel, plusieurs auteurs ont publié des résultats avec un recul moyen variable. Ainsi, Jones [13] a réévalué après 15 ans, 20 patients sur 27 du fait de décès, de complications sévères ou de perte de vue de patients. Dans la série de Serraf [3], après une période médiane de suivi de 13,3 ans, tous les survivants étaient en classe 2-3 de la NYHA à part 2 qui étaient en classe 3-4 de la NYHA. Moses [15] a signalé une amélioration fonctionnelle chez 19/26 patients, et l'absence d'aggravation chez le reste. Pour le devenir de l'IA, les conséquences de la levée chirurgicale du RASV sont très diversement appréciées. Certains auteurs décrivent une amélioration ou une stabilisation; d'autres au contraire une aggravation inéluctable après chirurgie. Marasini [5] n'a pas rapporté au suivi d'IA cliniquement significative et il l'a trouvée négligeable ou absente chez 26 des 44 patients avec une nette amélioration de l'IA en post-opératoire. Serraf [3] a constaté 49 cas d'amélioration de l'IA, 4 cas de stabilité, 2 cas d'aggravation et 21 cas d'apparition d'une IA de grade 1. Tous les autres n'ont pas eu d'aggravation de leur IA pendant la durée du suivi. Aucun des facteurs préopératoires testés (l’âge, le gradient préopératoire, la technique chirurgicale, l'anatomie et la fonction valvulaire préopératoire) n'ont influencé la fonction de la VA. Stellin [11], sur 55 IA préopératoires, a constaté 24 cas de régression, 31 cas de stabilité et sans aucun nouveau cas. Hansen [16], avec un recul moyen de 5ans après l'intervention, a rapporté l'apparition d'une IA chez 8 patients sur 13 qui n'avaient pas d'IA préopératoire. Limites de l’étude: Le caractère rétrospectif de notre série avec toutes les insuffisances rencontrées avec ce genre d’étude est surement un inconvénient de taille. Egalement, le nombre de patients étudiés est faible et ceci peut être en partie expliqué par le caractère militaire de notre principale origine de recrutement et l'aspect peu commun de la pathologie.

## Conclusion

La chirurgie du RASV et spécialement le DSA devient de plus en plus codifiée avec une tendance pour les techniques de plus en plus agressives vis-à-vis de la sténose et de la composante myocardique sous-jacente. Si les résultats de la chirurgie et le pronostic des formes localisées comme le DSA sont excellents, ceci est moins évident pour les formes étendues telle que le tunnel sous aortique et les anomalies de l'appareil mitral.

### Etat des connaissances actuelle sur le sujet

- C'est une chirurgie qui est de plus en plus codifiée avec des résultats en amélioration et une tendance vers une chirurgie de plus en plus préventive.

### Contribution de notre étude à la connaissance

- Notre travail montre l'expérience d'un centre africain avec une revue exhaustive de la littérature internationale.
